# Octahedral Molybdenum Iodide Clusters Supported on Graphene for Resistive and Optical Gas Sensing

**DOI:** 10.1021/acsami.2c15716

**Published:** 2022-12-13

**Authors:** Juan Casanova-Chafer, Rocio Garcia-Aboal, Pedro Atienzar, Marta Feliz, Eduard Llobet

**Affiliations:** † MINOS Research Group, Department of Electronics Engineering, Universitat Rovira i Virgili, Tarragona43007, Spain; ‡ Instituto de Tecnología Química, Universitat Politècnica de València - Consejo Superior de Investigaciones Científicas (UPV-CSIC), Avd. de los Naranjos s/n, Valencia46022, Spain

**Keywords:** molybdenum cluster, graphene, gas detection, resistive sensor, optical sensor

## Abstract

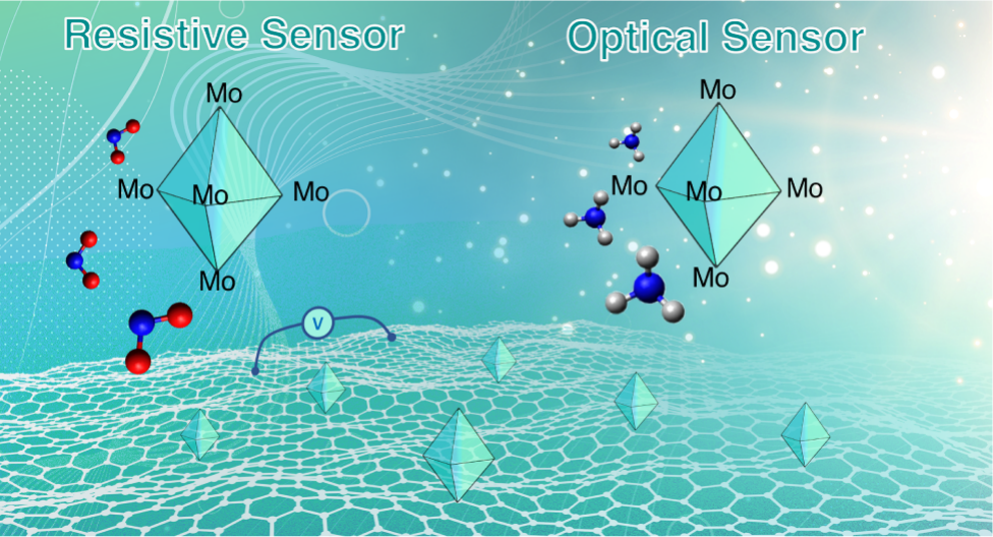

This paper reports for the first time a gas-sensitive nanohybrid based on octahedral molybdenum iodide clusters supported on graphene flakes (Mo_6_@Graphene). The possibility of integrating this material into two different transducing schemes for gas sensing is proposed since the nanomaterial changes both its electrical resistivity and optical properties when exposed to gases and at room temperature. Particularly, when implemented in a chemoresistive device, the Mo_6_@Graphene hybrid showed an outstanding sensing performance toward NO_2_, revealing a limit of quantification of about 10 ppb and excellent response repeatability (0.9% of relative error). While the Mo_6_@Graphene chemoresistor was almost insensitive to NH_3_, the use of an optical transduction scheme (changes in photoluminescence) provided an outstanding detection of NH_3_ even for a low loading of Mo_6_. Nevertheless, the photoluminescence was not affected by the presence of NO_2_. In addition, the hybrid material revealed high stability of its gas sensing properties over time and under ambient moisture. Computational chemistry calculations were performed to better understand these results, and plausible sensing mechanisms were presented accordingly. These results pave the way to develop a new generation of multi-parameter sensors in which electronic and optical interrogation techniques can be implemented simultaneously, advancing toward the realization of highly selective and orthogonal gas sensing.

## Introduction

Air pollution is probably the largest environmental health threat worldwide, accounting for 7 million premature deaths every year.^1^ Despite the fact that some techniques, such as gas chromatography coupled to mass spectroscopy, can selectively detect pollutants at trace levels, these instrumental techniques are costly, bulky, use consumables, and require frequent attention from qualified personnel. This is why automated air quality control stations are few and sparsely distributed in specific areas. However, effective pollution monitoring can only be achieved through a network of denser analysis locations (higher granularity) distributed over wider areas.^2^ In other words, the ubiquitous, unattended, and real-time detection of harmful gases would protect human health by allowing the taking of pertinent actions in space and time. In this perspective, the development of gas sensors with the ability to monitor low concentrations of flammable, combustible, or toxic gases in real time is crucial. These devices should gather essential properties such as reliability, sensitivity, selectivity, repeatability, accuracy, and long-term stability together with inexpensiveness and low power consumption.

Among the different technologies for developing gas sensors, chemoresistive devices have attracted great research efforts owing to their simple driving and readout instrumentation, durability, high potential for miniaturization, and industrial scalability.^3^ For decades, metal oxide (MOX) semiconductors have dominated the field of gas detection because of their high sensitivity, non-toxicity, and availability.^4^ However, their poor selectivity and power-hungriness (operation temperatures are high above room temperature) are still preventing their effective implementation in commercial applications for air quality monitoring stations or unattended gas sensing networks. Therefore, the development of new and energy-efficient gas-sensitive nanomaterials is still much needed.

In recent years, carbon nanomaterial-based sensors, especially those employing graphene, have become a common approach for developing devices that operate at room temperature.^5^ Room-temperature-operated gas sensors are energy efficient and help reduce fabrication costs (as no heating elements are needed), which makes them very attractive for being integrated into air quality monitoring networks. Despite the noteworthy properties of graphene, such as high surface area to volume ratios, low noise levels, high carrier density and mobility, and affordable production costs,^6^ there are still some issues to be overcome. Graphene, especially in its pristine form, shows limited sensitivity and selectivity. In this sense, large research efforts have been focused on modifying or functionalizing carbon-based films for improving their sensing properties. The most direct functionalization strategies are probably the modification of graphene via plasma treatments for creating defects and replacing carbon atoms with others of different nature,^7^ or the use of wet chemistry processes for the covalent or non-covalent functionalization of the graphene surface.^8^ With these approaches, the sensitivity of the graphene layers is generally improved, but usually the selectivity issue remains only partially addressed, owing to the poor specificity of the atoms and functional groups present on the graphene surface. Another well-known strategy is the functionalization of graphene with organic compounds such as macrocycles, aptamers, or cavitands.^9,10^ This approach significantly improves the selectivity because of the high specificity of the organic compounds (i.e., acting as chemical receptors) toward specific gases. However, the organic nature of this functionalization, which is prone to degradation under operational conditions, may compromise long-term device stability. Additionally, high specificity may result in limited reversibility of the gas–solid interactions, which compromises response repeatability. Finally, the last mainly used strategy is the decoration of graphene with metal or MOX nanoparticles.^11,12^ With that, it is possible to significantly increase the sensitivity and tune the selectivity to some extent. However, these MOX nanoparticles usually need moderate or high operating temperatures for activating their sensing properties. Therefore, the achievement of low-power and inexpensive devices can be jeopardized.

As an alternative, molybdenum chalcogenides and their derivatives have been extensively explored for gas detection in the last few years.^13−15^ These nanostructured semiconductors vary in composition and morphology, which impacts their sensing performance.^16,17^ Besides, the fabrication of molybdenum sensors is attractive due to their abundance and low toxicity, the development of thin films and nanoparticles being the most common strategy.^18^ However, there is significant scope remaining to explore new molybdenum materials with advanced properties for large-scale fabrication. Particularly, materials composed of architectured nanometer-sized octahedral molybdenum clusters (<2 nm) are very attractive for gas sensing purposes.^19^ These [Mo_6_L^i^_8_L^a^_6_] cluster units are robust entities composed of inner halide ligands (L^i^) and organic or inorganic apical (or terminal) ligands (L^a^). Mo_6_ clusters act as powerful photosensitizers and are also efficient luminophores that, under photoactivation, either exhibit phosphorescence in the 550–900 nm window or react with oxygen to provide singlet molecular oxygen (^1^O_2_) directed to cell damage by oxidative stress and to theranostic applications. Until now, the existing literature on analyte sensing using Mo_6_ cluster-based nanomaterials encompasses optical oxygen sensors,^20−24^ optical sensing of antibiotics and nitroaromatic compounds in solution,^25,26^ and cluster dependence on environmental parameters, such as humidity or light.^27,28^ However, Mo_6_ clusters have not been applied to the detection of air pollutants until now, and the research of the materials composed of Mo_6_ clusters and graphene and graphene oxide was limited to the photocatalytic field.^29−32^

To the best of our knowledge, the use of hexanuclear molybdenum cluster nanomaterials for the detection of pollutant species in the gas phase remains unexplored. Therefore, this paper encompasses, for the first time, the preparation of a Mo_6_@Graphene nanohybrid composed of a crystalline octahedral molybdenum iodide cluster material supported on graphene, and the integration of this hybrid onto a transducing platform, enabling chemoresistive and optical gas sensing. The detection of two gaseous species such as nitrogen dioxide (NO_2_) and ammonia (NH_3_) is studied. NO_2_ is an electron-withdrawing compound mainly released by automotive emissions and combustion of conventional fossil fuels, which has become a major environmental concern because it contributes to the formation of photochemical smog, acid rain, and particulate matter through chemical reactions in the atmosphere. Ammonia emissions are associated with industrial livestock, fertilizer production processes, and crop agriculture. As a highly toxic and corrosive agent, they threatenhuman health and the environment.^33^ Besides reporting the sensing properties of the Mo_6_@Graphene hybrid nanomaterial, its thorough morphological and compositional characterization is reported as well. Finally, the gas sensing mechanisms involved in the chemoresistive and optical detection are presented and discussed.

## Results and Discussion

### Synthesis and Characterization of the Nanomaterials

The crystalline [Mo_6_I^i^_8_(OH)^a^_4_(H_2_O)^a^_2_]·2H_2_O (Mo_6_) material and its derived nanohybrid (Mo_6_@Graphene) were prepared and analyzed using several techniques such as Raman spectroscopy, powder X-ray diffraction, field-emission scanning electron microscopy (FESEM), and high-resolution transmission electron microscopy (HR-TEM). The synthesis of Mo_6_ was achieved by the hydrolysis of (Bu_4_N)_2_[Mo_6_I^*i*^_8_(O_2_CCH_3_)^*a*^_6_].^29^ The immobilization of Mo_6_ on graphene was done by mixing the respective precursors dispersed in dichloromethane and confirmed through Raman spectroscopy (Figure S1a), resulting in Mo_6_@Graphene. In this regard, the Raman shifts below 360 cm^–1^ are associated with the presence of molybdenum clusters present in the Mo_6_ material (Figure S1b), while at higher frequencies, the characteristic D, G, 2D, and D′ bands of graphene appear at 1347, 1588, 2712, and 2938 cm^–1^, respectively. The D band is associated with the presence of structural defects, disordered carbon atoms in the sp^2^ configuration, and carbonaceous impurities, while the G band is related to in-plane vibrations of sp^2^ carbon bonds. Thereby, the D/G ratio revealed low crystalline graphene, and the presence of defects and oxygenated functional groups grafted to the graphene is probably supporting the immobilization of molybdenum clusters. The immobilized inorganic clusters at the graphene surface are essential in gas sensing performance, enabling higher interactions with gas compounds. Furthermore, the integrity and crystallinity of the cluster-based materials after the thermal treatment (60 °C) needed for the preparation of the resulting Mo_6_@Graphene were studied. Figure S1b shows intense bands with Raman shifts at 129, 153, 292, and 357 cm^–1^ associated with the Mo–Mo, Mo–I, and Mo–O vibrations of the cluster.^29,34^ These Raman shifts are similar to those reported for similar hexamolybdenum clusters with iodide materials, revealing that the thermal treatment applied during the experimental protocol does not damage the cluster crystallinity owing to the lack of differences between both spectra. The suitable cluster synthesis and integrity after the thermal treatment were also confirmed through powder X-ray diffraction. Figure S2a depicts the diffractogram of the Mo_6_ material, which shows a family of planes associated with a preferential orientation of the crystalline material upon deposition onto the glass surface.^29^ The Mo_6_ crystallinity was preserved after the thermal process applied during the deposition step (Figure S2b). Figure S2c shows the diffractogram of the Mo_6_@Graphene after the deposition process. Considering the low graphene crystallinity, it is noteworthy that some intense peaks associated with the crystallinity of Mo_6_ can be noticed in the nanohybrid, evidencing the presence of supported cluster crystals. Thereby, the X-ray diffraction also confirms that the synthesis and deposition processes developed avoided the degradation of molybdenum cluster-based material. The robustness of the prepared cluster materials is an advantage for preparing gas sensors and conducting the resistive and optical measurements described below. Regarding the binding interaction between the crystalline cluster phase and the graphene sheets, we think that it could mainly be associated with supramolecular interactions, such as Van der Waals and charge electrostatic and polar interactions, due to the polarity induced by the cluster units and the presence of water molecules embedded into the hydrogen-bonded network of the crystalline cluster phase and the preference of such water molecules to adsorb on residual defects and sites with oxygen-containing functional groups.^30,35,36^ However, the cluster immobilization of {Mo_6_I^i^_8_}^4+^ cluster cores through coordinative bonds to such oxygen functionalities, as occurs with GO, cannot be discarded.^29^ Additional research on the study of the binding interactions involved is under current investigation.

**Figure 1:**
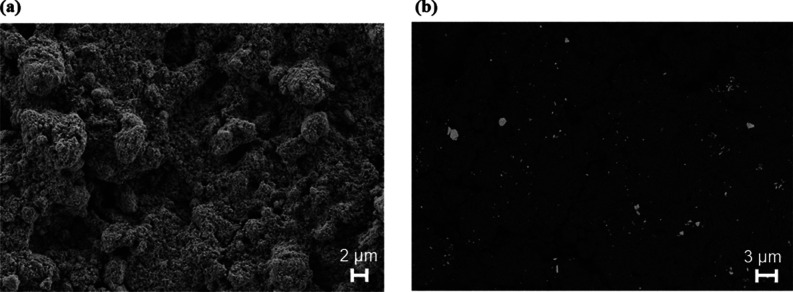
(a) FESEM image showing the sensor surface of bare graphene. (b) FESEM image recorded with the BSE detector of the Mo_6_@Graphene material. The {Mo_6_I^i^_8_}^4+^ cluster core material (bright spots) supported on graphene (dark background) can be observed, revealing a suitable cluster distribution.

Finally, the resulting Mo_6_@Graphene nanomaterial was analyzed by FESEM and HR-TEM techniques. Figure 1a shows the graphene surface once it was deposited. A highly porous surface can be noticed (BET area of 730 m^2^/g according to previous results^37^), which can be interesting from the sensing point of view. Moreover, Figure 1b confirms the well-dispersed crystalline cluster particles supported on graphene. This image was taken employing a back-scattered electron (BSE) detection; consequently, bright spots correspond to Mo_6_ and the dark background corresponds to graphene. HR-TEM and energy-dispersive X-ray spectroscopy (EDS) analyses of the nanomaterial confirm the presence of the Mo_6_I^i^_8_ cluster units deposited onto graphene layers (Figure S3).

### Gas Detection Using a Chemoresistive Transducing Scheme

**Figure 2:**
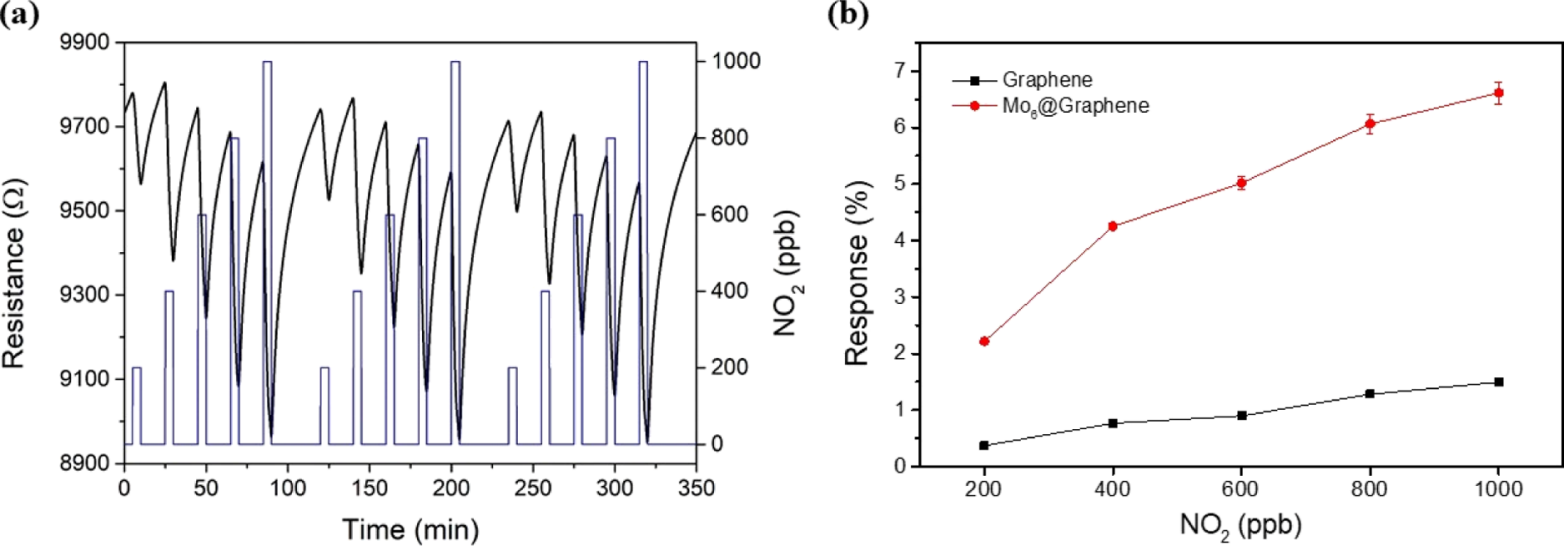
(a) Electrical responses toward NO_2_ (range 250–1000 ppb) under room temperature operation using the Mo_6_@Graphene nanomaterial. (Resistance: black line and left *y*-axis; gas concentration: blue line and right *y*-axis). (b) Comparison of the calibration curves obtained for bare graphene and Mo_6_@Graphene for detecting NO_2_.

The ability of the Mo_6_@Graphene layer for detecting several gas species was evaluated under room temperature operation conditions. This approach leads to low power-consumption devices and reduced fabrication costs. Different concentrations (250, 500, 750, and 1000 ppb) of NO_2_ were applied during several consecutive measurement cycles (Figure 2a), obtaining significant differences when graphene is loaded with molybdenum clusters in comparison to its bare counterpart (Figure S4). Specifically, the calibration curves (Figure 2b) revealed that the Mo_6_@Graphene sample presents up to fivefold higher electrical responses (i.e., the intensity of the resistance changes induced by the exposure to NO_2_) than bare graphene. Regarding the sensitivity, which is given by the slope of the curves shown in Figure 2b, the Mo_6_@Graphene layer shows 4 times higher sensitivity toward NO_2_ than bare graphene.

**Figure 3:**
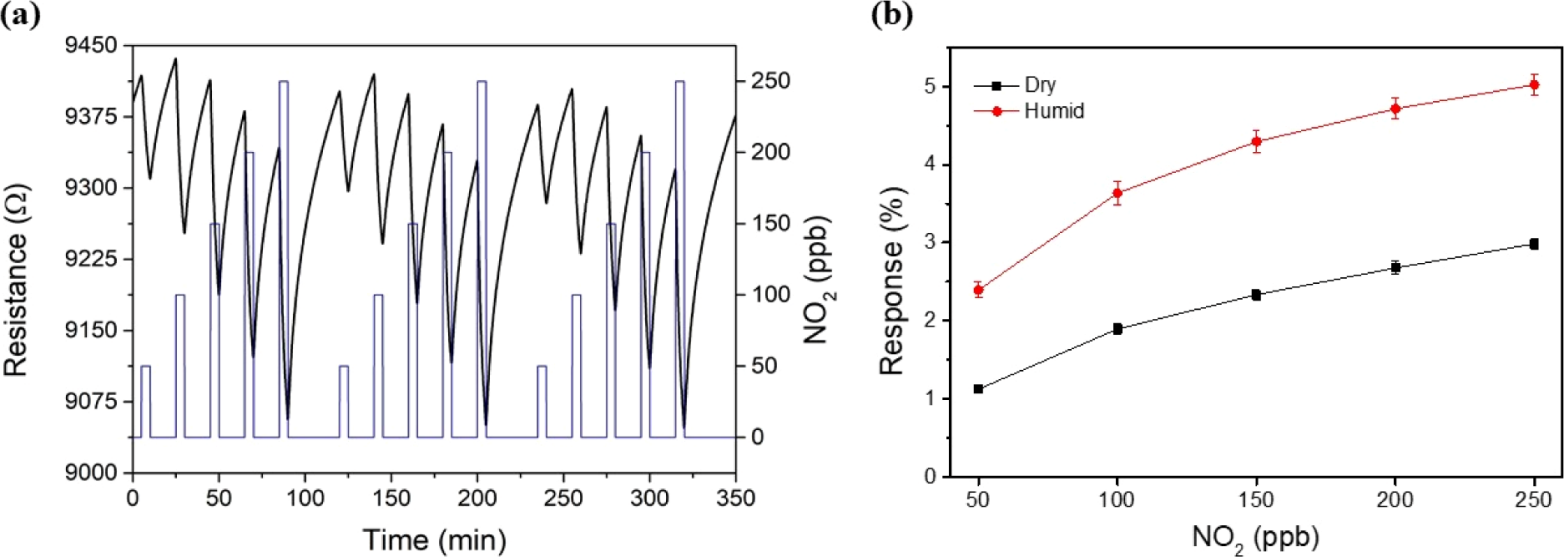
(a) Dynamic response toward NO_2_ (range 50–250 ppb) under room temperature operation for the Mo_6_@Graphene nanocomposite. (Resistance: black line and left *y*-axis; Gas concentration: blue line and right *y*-axis). (b) Comparison of the calibration curves for the Mo_6_@Graphene layer in a dry and humid atmosphere.

Considering the requirements for ambient monitoring purposes, the detection of lower NO_2_ concentrations is needed, and the effect of the relative humidity should also be assessed owing to its significant effect on sensing performance. Thereby, according to the threshold limit values (TLV) defined by the Clean Air Act Text (the United States Environmental Protection Agency) and the Ambient Air Quality (European Union),^38,39^ lower NO_2_ concentrations ranging from 50 to 250 ppb were tested. Figure 3a shows an intelligible and repeatable detection of the target gas by the Mo_6_@Graphene layer, enabling its potential use in commercial applications. It is worth noting that, even at this low range of NO_2_ concentrations, the Mo_6_@Graphene sensor shows good baseline stability under room temperature operation conditions. Conversely, bare graphene presents a poor sensing performance at this concentration range (Figure S5a), experiencing low repeatability due to the slight resistance changes induced by the exposure to the target gas.

Not limited to this, since ambient moisture is a well-known interfering element on the gas sensing performance,^40^ NO_2_ ranging from 50 to 250 ppb was also tested under humid conditions (Figure S5b). In consequence, the previous experiment carried out under dry conditions was reproduced at 60% relative humidity for the cluster-loaded graphene. Figure 3b depicts a comparison for the Mo_6_@Graphene sample in both environments, revealing a significant increase in the sensing response (up to 2-fold) when the sensor is operated under humid conditions. However, the sensitivity (i.e., the slope of the calibration curve) remains almost unchanged under dry or humid conditions, which is an outstanding result toward implementation in ambient monitoring devices.

In view of the intelligible sensing responses and the low noise levels registered, the limits of detection and quantification (LOD and LOQ, respectively) were evaluated measuring trace levels of NO_2_ ranging from 10 to 25 ppb (Figure S6a). The calibration curve (Figure S6b) was used for estimating both parameters through the following equations
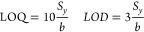
where *S*_*y*_ are the standard deviations of *y*-residuals, and *b* corresponds to the sensitivity (slope) of the calibration curve. Besides, a factor of 10 and 3 is applied to the LOQ and LOD, respectively. As a result, the Mo_6_@Graphene sensitive layer reveals a LOQ of 10.3 ppb, while the estimated LOD is 3.1 ppb. It is worth noting that these values are far below the TLV needed for ambient monitoring applications, and the detection times are enough for enabling a reliable detection considering that the exposure limits are usually defined as the average concentrations for 8 h. The device operating conditions (i.e.*,* 5 min of gas exposure and room temperature working conditions) result in advantageous features such as low power consumption, high sensitivity, and fast detection of gas species.

**Figure 4:**
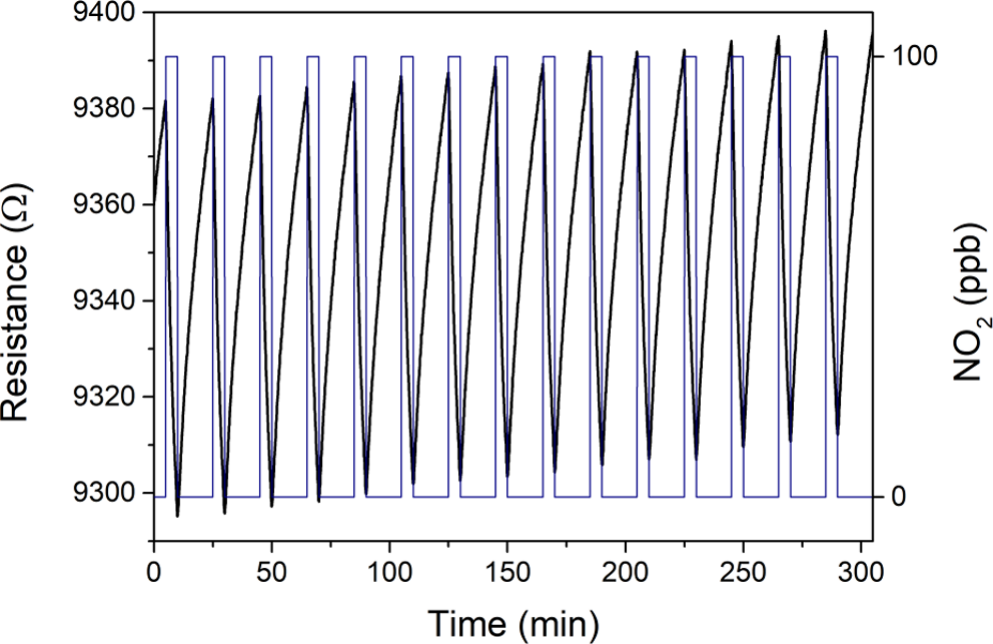
Repeatability experiment for the Mo_6_@Graphene device by applying 15 pulses of 100 ppb of NO_2_. (Resistance: black line and left *y*-axis; Gas concentration: blue line and right *y*-axis).

Gas sensor repeatability is a key influencing parameter that determines the potential of the developed cluster-graphene layer for being employed in gas sensing. In consequence, successive cycles of 5 min of NO_2_ exposure followed by 15 min of recovery in dry air were recorded (Figure 4). As a result, the hybrid Mo_6_@Graphene revealed outstanding sensor repeatability, showing an error of about 0.9%.

**Figure 5:**
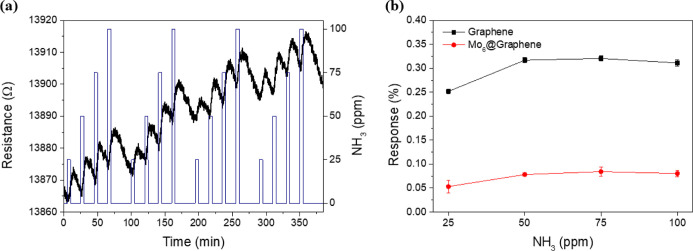
(a) Example of the dynamic responses for the Mo_6_@Graphene sensor when detecting NH_3_ under room temperature conditions. (Resistance: black line and left *y*-axis; Gas concentration: blue line and right *y*-axis). (b) Comparison of the calibration curves obtained for bare graphene and Mo_6_@Graphene for detecting NH_3_.

Apart from the detection of an electron-withdrawing gas like NO_2_, the sensing performance of bare and cluster-loaded graphene was also evaluated toward an electron-donor species as ammonia (NH_3_). Figure 5a shows the resistance changes obtained for the Mo_6_@Graphene sample when detecting NH_3_ in the range of 25–100 ppm. A significant baseline drift can be observed, probably because the sensor surface is not fully cleaned and some NH_3_ is still adsorbed. This behavior is relatively frequent when gas sensors are operated at room temperature and under low flow rates. Nevertheless, baseline drift might be ameliorated by irradiating the surface with UV light or slightly increasing the operating temperature, only to cite some strategies. Indeed, the chemoresistive response for bare graphene (Figure S7) is higher than that of cluster-loaded graphene, as Figure 5b depicts. Nevertheless, considering that sensitivity is given by the slope of the calibration curve, it can be concluded that Mo_6_@Graphene is virtually insensitive to NH_3_; since responses are lower than 0.1%, these barely change when exposed to different ammonia concentrations. It should be mentioned that NH_3_ sensing performance might be improved to some extent, but the same experimental conditions were applied as NO_2_ for an effective comparison.

In view of these results, it was necessary to check whether or not the absence of a chemoresistive response for ammonia could be due to the decomposition of {Mo_6_I^i^_8_}^4+^ cluster cores, owing to the basicity of NH_3_.^41^ In this perspective, the Mo_6_ and Mo_6_@Graphene robustness upon exposure to NO_2_ and NH_3_ gases was evaluated by powder X-ray diffraction and Raman analyses. Figure S8 shows X-ray diffractograms for both samples before and after being exposed to the target gases. Since no change in the spectra is observed, the gas exposure does not damage the cluster crystallinity. Accordingly, the Raman spectra confirm that the cluster units are not decomposed after exposure to gases (Figure S9). This reveals the outstanding stability of the molybdenum cluster units, which are not degraded when exposed to ammonia or nitrogen dioxide.

### Gas Detection Using an Optical Transducing Scheme

**Figure 6:**
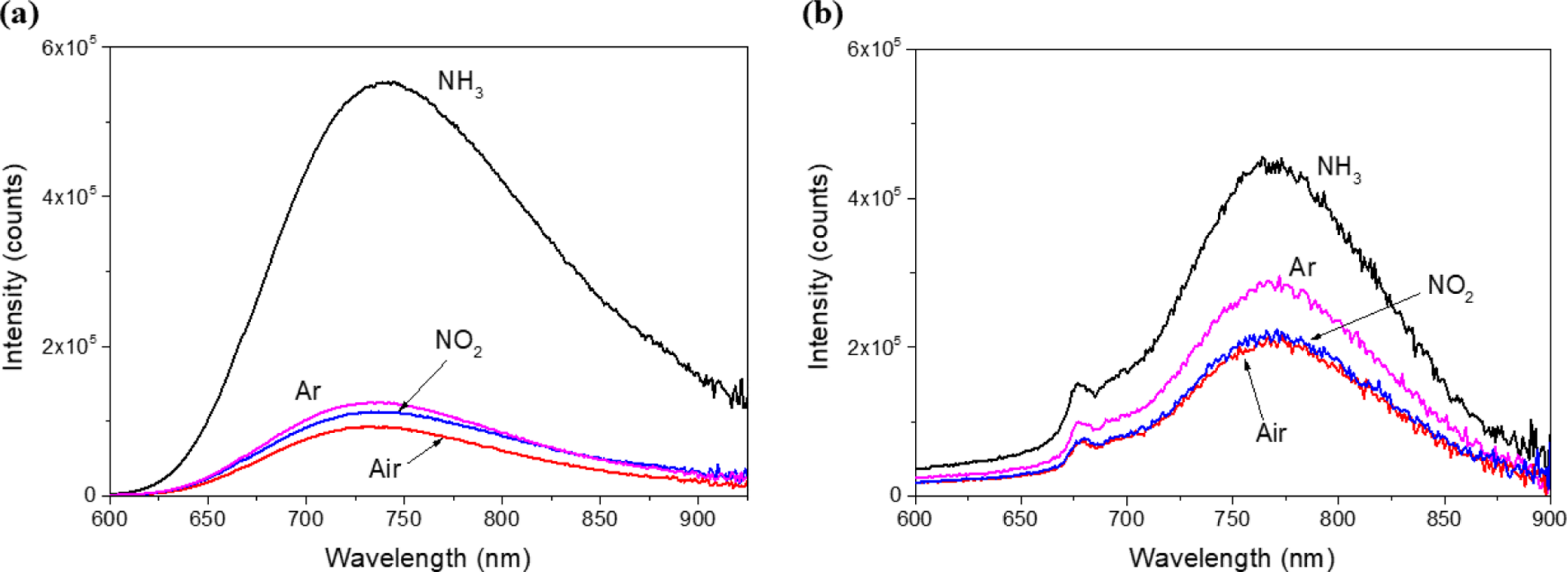
(a) Room-temperature PL spectra of a thin film of pure Mo_6_ material deposited in a quartz substrate (λ_ex_ = 320 nm) and exposed under Ar, air, NO_2,_ and NH_3_ gases. (b) Room-temperature PL spectra measured directly onto Mo_6_@Graphene coating on the alumina substrate (λ_ex_ = 320 nm), employed in chemoresistive transduction, and exposed under Ar, air, NO_2,_ and NH_3_ gases.

The optical gas sensing properties of the Mo_6_@Graphene and Mo_6_ materials were preliminarily investigated using films deposited on alumina and quartz transducer substrates, respectively, and in the presence of NO_2_ (1 ppm) and NH_3_ (250 ppm) balanced in air. Photoluminescence (PL) spectroscopy provides information about the interaction between molybdenum clusters and target gases. The steady-state emission spectrum of the pure Mo_6_ crystalline material presents a broad emission band centered at 725 nm when registered under an argon atmosphere (Figure 6a). This wavelength is characteristic of the electronic triplet excited state of the{Mo_6_I^i^_8_}^4+^ cluster core.^42−46^ A similar spectrum was registered for the Mo_6_@Graphene device under an inert atmosphere (Figure 6b), but a redshift to 770 nm was detected with no interference of the characteristic emission band of graphene (Figure S10). This shift of the PL for the Mo_6_@Graphene material corresponds to a band gap decrease of 0.1 eV, and it can be attributed to two main factors: the polarity conferred by the graphene environment and the interactions between defective graphene and Mo_6_ material. Both films were exposed to NO_2_, NH_3,_ or O_2_ (air) gases, and the PL changes were measured. In their in situ optical characterization, intensity variations were observed in the emission spectra with respect to the analyte gases. A decrease in the PL was observed in the presence of air with respect to exposure under argon. This is due to efficient PL quenching by oxygen, leading to the formation of singlet molecular oxygen (^1^O_2_), which is an intrinsic property of the {Mo_6_I^i^_8_}^4+^ cluster core materials.^47^ A decrease in the PL was also observed after exposure to NO_2,_ and this PL quenching is achieved to a lesser extent than in the presence of air. In contrast, a high PL was detected in the presence of ammonia. The different optical responses of the molybdenum crystalline material with the NH_3_ and NO_2_ gases follow the same trend for the Mo_6_@Graphene nanomaterial. The PL was recorded after two consecutive NH_3_/Ar cycles, and the results indicated the reversibility of the PL responses (Figure S11). These surprising results indicate that “wake-up” ammonia detection of the cluster-based Mo_6_ and Mo_6_@Graphene materials is achieved. Apart from the donor/acceptor properties of the gas molecules, these phenomena could be associated with a strong interaction between NH_3_ and the molybdenum clusters. Whereas the Mo_6_ material shows high sensing performance and selectivity toward ammonia detection, only the hybrid material shows a selective double transduction scheme (optic and chemoresistive). In addition, a remarkable PL response for the Mo_6_@Graphene hybrid was obtained considering the low concentration of supported clusters. A possible quenching effect of the graphene support can also be responsible for the lower selectivity toward gas analytes.^30^ The stability of the Mo_6_ and Mo_6_@Graphene materials when exposed to ammonia or nitrogen dioxide supports the reliability of the PL spectra. Since the emission bands are exclusively associated with the luminescence of the octahedral Mo clusters, the viability of both nanomaterials as optical and chemoresistive sensors was confirmed.

### Orthogonal Sensing Mechanisms

The above results show that the Mo_6_@Graphene film coating over standard substrates can be interrogated using two different transducing schemes, chemoresistive and optical, for the detection of nitrogen dioxide and ammonia, respectively. This behavior would correspond to orthogonal sensing, which is a term coined in the context of detecting different toxic gases or even multi-component gas mixtures using sensor arrays. Ideally, orthogonal sensing would be achieved because at least one highly specific sensor is present in the array for every target species. In a real application, orthogonality can be achieved either by modifying/optimizing the gas-sensitive film to make it more specific to its target species (in this approach, specificity or selectivity are in general improved to a limited extent) or by constructing orthogonal metasensors built upon the combination of response features from an array of sensors with partially overlapped selectivity.^48,49^ The resistive and optical results described in this work pave the way for devising a non-conventional strategy for achieving orthogonal sensing.

**Figure 7:**
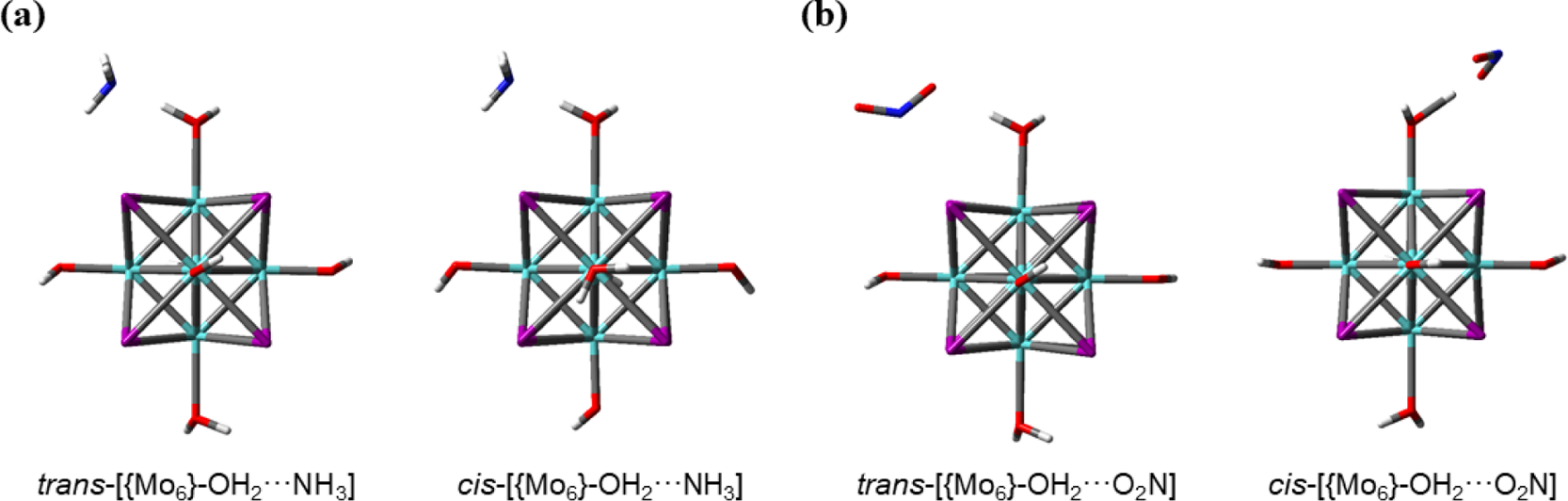
Representation of the most stable cluster···NH_3_ (a) and cluster···NO_2_ (b) models, encompassing *trans-* and *cis*-[Mo_6_I^i^_8_(OH)^a^_4_(H_2_O)^a^_2_] configurations (right and left representations, respectively).

The factor responsible for this orthogonal behavior is related to the interaction between the target gas analytes and the molybdenum cluster-based material. The integrity of the Mo_6_@Graphene and Mo_6_ materials after the exposure to gases confirms that, even considering the coordinative ability of ammonia, no ligand exchange over the {Mo_6_I^i^_8_}^4+^ cluster units was achieved. This suggests that the gas–solid interaction would take place by other kinds of supramolecular interactions, hydrogen bonding being the most plausible one. In the case of transition metal luminophores, the hydrogen bonding interactions with polar ligands, which act as receptor sites capable to form hydrogen bonds with the target molecule, can provide changes in their characteristic luminescence response.^50−54^ Since the X-ray diffraction planes of the deposited Mo_6_ material suggest that the [Mo_6_I^i^_8_(OH)^a^_4_(H_2_O)^a^_2_] cluster sites are preferentially exposed to gas adsorption, the sensing is probably promoted by hydrogen bonding between the apical cluster ligand (hydroxo or water) and the gaseous analytes. To reveal the interactions between the [Mo_6_I^i^_8_(OH)^a^_4_(H_2_O)^a^_2_] clusters and target molecules, DFT calculations were performed using the BPE0 functional because of its robustness and excellent performance for treating hydrogen bonds.^55,56^ Two stereoisomers, namely *trans* and *cis*-[Mo_6_I^i^_8_(OH)^a^_4_(H_2_O)^a^_2_], were built as representative models for adsorption sites, and the reliability of this functional was confirmed by the analysis of the computed geometries of the isomers, which shows a good fit to the X-ray diffraction structure (Figure S12). The energetic interactions between the cluster compound and the gas molecules were studied by computing the binding energies (BEs) associated with the formation of a cluster-gas molecule (NH_3_ or NO_2_) adduct in a 1:1 ratio. In all the adducts, the gas interacts directly with water and hydroxo ligands (Figures 7 and S13) by hydrogen bonding in most cases, with BEs in good agreement to H-bonding energies (Table S1). Figure 7 illustrates the most energetically stable cluster-adduct models with the highest BEs, namely *ca.* −18 and −7 kcal/mol, associated with the isomers with H_2_N···HOH and NO_2_···HOH interactions, respectively. The gas-cluster adducts become energetically more unstable when the hydroxo ligand is involved (Table S1). In all cases, BEs are negative, indicating that the formation of cluster-gas molecule adducts is exothermic. Thus, NH_3_ and NO_2_ molecules are likely to be adsorbed onto the surface of the cluster-based crystalline material.

**Figure 8:**
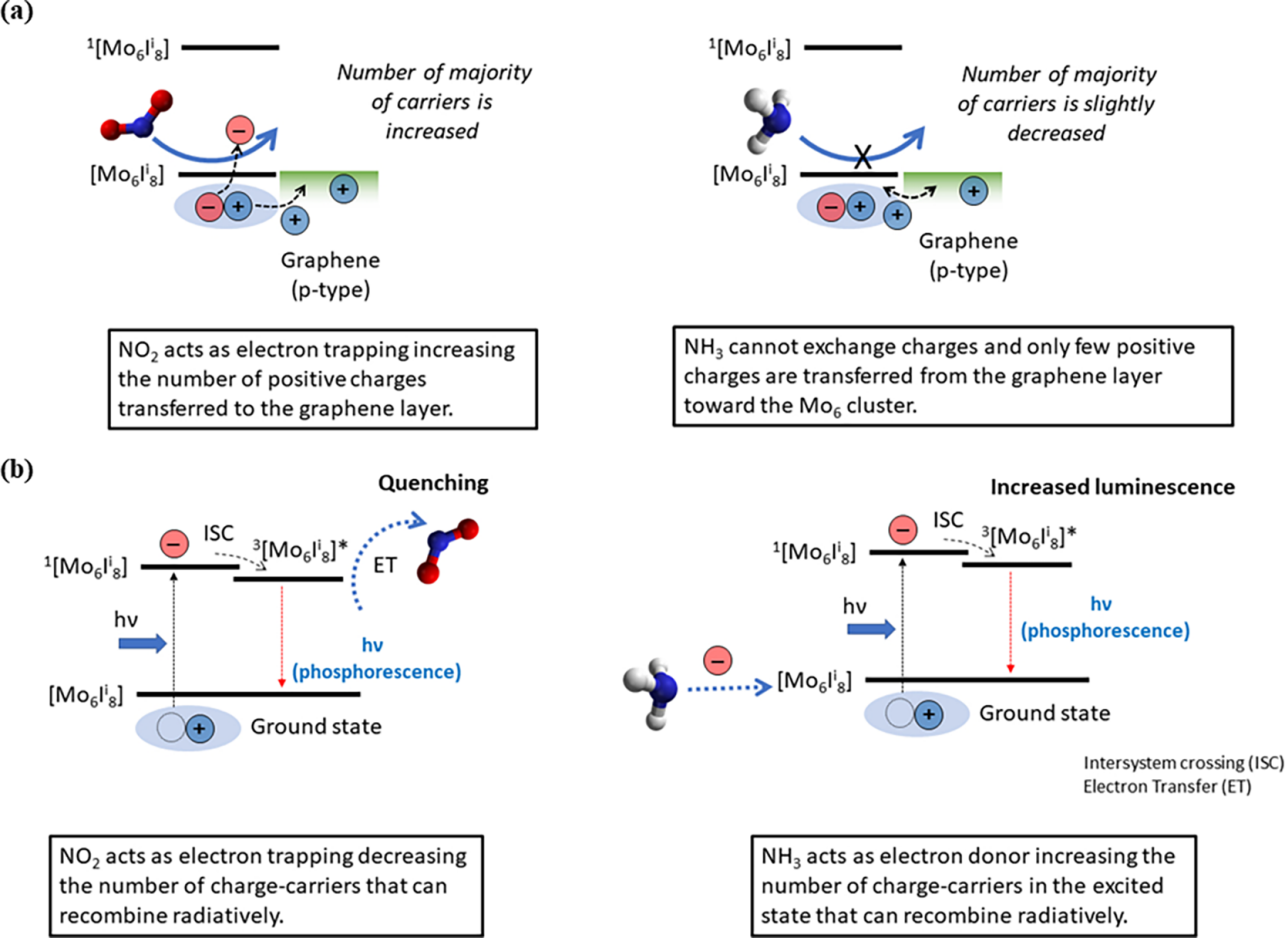
Schematic representation of the two sensing mechanisms proposed under resistive (a) and optical (b) conditions. The graphs on the left and right correspond to NO_2_ and NH_3_ gas exposure, respectively.

A tentative description of the sensing mechanism of [Mo_6_I^i^_8_(OH)^a^_4_(H_2_O)^a^_2_] toward NH_3_ and NO_2_ under resistive and optical conditions is represented in Figure 8. Under resistive conditions (Figure 8a), the exposure of the molybdenum clusters to the electron-acceptor NO_2_ would favor the increase of positive charges in graphene layers, resulting in a major chemoresistive response. In the presence of NH_3_, its electron donor ability practically does not influence the electronic characteristics of the clusters, and, as a consequence, they would remain intact to exchange charges with graphene. This could be associated with the difficulty in the reduction of the {Mo_6_I^i^_8_}^4+^ cluster core compounds, considering their intrinsic redox properties.^57^ One question remains open regarding the small chemoresistive response of Mo_6_@graphene in the presence of ammonia in comparison with one of the bare graphenes (Figure 5). Probably, this behavior can be explained considering the close alignment of the energy bands between the fundamental state of the octahedral molybdenum clusters and the work function of graphene. The value of the work function of graphene is dependent on the oxygen functionalities (between 4.2 (pristine graphene) and 6.7 eV),^58^ and the functionalization onto the graphene surface.^59^ The positioning of the fundamental state of {Mo_6_I^i^_8_}^4+^ cluster core compounds (*ca.* 5.0–5.5 eV)^29,30^ would promote the transfer of some holes from the graphene layer to the metallic cluster and concomitant compensation of charges, giving rise to a diminution of the resistive response.

Under light irradiation conditions (Figure 8b), the exposure to NH_3_ gas molecules, which act as electron donors, would increase the concentration of charges in the fundamental state of {Mo_6_I^i^_8_}^4+^ cluster core molecules, and a huge increase in the PL intensity is detected. In contrast, the interaction of the electron acceptor NO_2_ molecules with the molybdenum clusters would only slightly quench the PL in an electron transfer process, similar to that described for O_2_. Probably, these photophysical effects can explain the orthogonal behavior of the Mo_6_@Graphene sensor under electrical conditions. The great PL increase in the presence of NH_3_ gas could promote charge recombination by emitting light and hinder charge transfer with graphene, resulting in poor resistive changes. However, the electronic structure of molybdenum clusters is practically unaffected under NO_2_ gas and remains intact to exchange charges with graphene.

## Conclusions

A novel nanomaterial based on molybdenum iodine clusters supported on graphene was implemented in a single gas sensing device, which employs two transduction schemes, that is, chemoresistive and optical. Noteworthily, the developed hybrid nanomaterial can work under room temperature conditions and can be easily integrated into miniaturized devices. Additionally, the crystalline material based on {Mo_6_I^i^_8_}^4+^ cluster cores integrated into graphene layers showed outstanding stability and low cross-sensitivity to ambient moisture. This approach has enabled the orthogonal detection of two gaseous species (i.e., NO_2_ and NH_3_) since nitrogen dioxide induces a chemoresistive response and not a PL response, while ammonia induces a PL response and not a chemoresistive response. DFT calculations confirmed that the gas sensing mechanisms are based on hydrogen bonding interactions between the analyte and the apical cluster water ligands of the octahedral cluster entities. Different mechanisms of charge transfer between {Mo_6_I^i^_8_}^4+^ cluster core molecules and the gas molecules are proposed for elucidating the chemoresistive response. The changes in the cluster emission are attributed to a change in the energy transfer efficiencies promoted by hydrogen bonding between the apical cluster ligands and NH_3_. These proof-of-concept results pave the way for developing new, highly selective, and multi-parameter gas sensors by combining electronic and optical transduction schemes in a single device. In consequence, Mo_6_@Graphene sensors represent an interesting alternative for achieving widespread air quality monitoring systems owing to their ease of use, inexpensiveness, low energy consumption, and outstanding gas sensing properties.

## Materials and Methods

### Synthesis of [Mo_6_I^i^_8_(OH)^a^_4_(H_2_O)^a^_2_]·2H_2_O (Mo_6_)

The synthesis of Mo_6_ microcrystals was achieved by optimizing a described procedure^29^ from the cluster precursor (Bu_4_N)_2_[Mo_6_I^i^_8_(O_2_CCH_3_)^a^_6_]. This was prepared by properly adapting the synthetic procedures reported in the literature from the (Bu_4_N)_2_[Mo_6_I^i^_8_I^a^_6_] and silver acetate materials.^44,60,61^ In a typical procedure for preparation of Mo_6_, the cluster precursor (30 mg) was dispersed in a mixture (15 mL) of water, acetone, and triethylamine in 50/45/5% v/v, respectively, in a round-bottom flask with continuous magnetic stirring (500 rpm) at room temperature. Afterward, a bubbler was connected to the flask, and the resulting yellow-orange suspension was heated to 35 °C for 1 h. Over time, the mixture evolved to a solution, which was added into a 10 mL vial sealed with a holey parafilm for its slow evaporation for 5–7 days. Finally, red microcrystals were collected from the remaining solution. The crystalline material was washed twice with acetone and once with Milli-Q water (10 mL). Notably, the optimum crystal size is achieved when the solution becomes transparent.

### Mo_6_@Graphene Development and Sensing Device Fabrication

For supporting the molybdenum clusters on graphene, two solutions were prepared in parallel. First, a 10 mL graphene suspension (0.5 mg/mL) in dichloromethane was performed employing graphene nanoplatelet aggregates (submicron particles, surface area 750 m^2^/g, Strem Chemicals Inc., USA). Then, the resulting suspension was placed in an ultrasonic tip for achieving a proper graphene exfoliation. Particularly, a pulsed sonication (1s on/2s off) at 280 W for 90 min was applied. A 1 mL suspension of Mo_6_ (0.25 mg/mL) in dichloromethane was prepared by sonication for 30 min in an ultrasonic bath. Afterward, the Mo_6_ mixture was added to the graphene suspension, resulting in graphene loaded with a 5% wt of the Mo_6_ cluster. The mixture was homogenized for 10 min under stirring. Subsequently, the solution was sonicated in an ultrasonic bath for 1 h, resulting in a suitable distribution of molybdenum clusters supported on graphene (Mo_6_@Graphene). For the characterization of the nanomaterial, the resulting suspension was deposited onto appropriate wafers directed to each characterization technique and placed onto a hotplate at a moderate temperature (60 °C). For sensing studies, the suspension was deposited via spray coating on alumina substrates comprising platinum screen-printed electrodes (Figure S14). First, the substrates were placed onto a hotplate at a moderate temperature (60 °C), and then, the deposition process was started after few minutes for ensuring the correct heating up of the substrate. The spray coating deposition was performed using nitrogen as the carrier gas for about 45 s. As a result, homogeneous sensitive films were obtained with an average thickness of 1 μm (see cross-section, FESEM (Figure S15) and optical profilometer measurement (Figure S16)).

### Characterization Techniques

UV–vis spectra were acquired at 20 °C, employing a Varian Cary 50 Conc spectrophotometer equipped with 10 × 10 mm quartz cells. Steady-state PL measurements were recorded in an Edinburgh Instruments FLS1100 spectrofluorometer using a 450 W xenon lamp light equipped with a double monochromator for excitation and emission coupled to a cooled photomultiplier (PMT-980). The Raman spectra were obtained from solid samples previously deposited onto quartz substrates using a “Reflex” Renishaw spectrometer equipped with an Olympus microscope. The exciting wavelength was 514 nm of an Ar^+^ ion laser, while the laser power was ∼10–25 mW; 20 acquisitions were taken for each spectrum. Powder X-ray diffraction patterns were obtained by using a Philips X′Pert diffractometer and copper radiation (CuKα = 1.541178 Å). FESEM images were recorded with a Zeiss Ultra 55 field FESEM apparatus (Atlanta, GA, USA) equipped with a BSE detector. Samples for HR-TEM were deposited onto carbon-coated copper grids. HR-TEM images were recorded by using a JEOL JEM2100F microscope operating at 200 kV. The molybdenum and iodine contents of the Mo_6_@Graphene sample were determined by dark-field scanning transmission electron microscopy (STEM) EDS analysis conducted by using an EDAX system (Oxford Instruments) attached to a JEOL JEM2100F electronic microscope. The thickness of the film was measured with a MicroXAM-100 3D surface profilometer. The cross-section was examined by FESEM (Zeiss Ultra 55 instrument).

### Resistive Gas Sensing Measurements

Once homogeneous layers were achieved (Figure S14), the gas sensors were placed in an airtight testing chamber (dead volume of 35 cm^3^). The chamber was subsequently connected to a gas mixing and delivery system comprising different calibrated gas cylinders. A pure dry air atmosphere (air premier purity: 99.999%) was employed during the sensing test and used as a carrier gas for the target gases. It is worth highlighting that a residual amount of relative humidity (3–4%) is present in dry conditions. The overall flow was adjusted at a low rate (100 mL/min) using a set of mass-flow controllers (Bronkhorst High-Tech B.V., The Netherlands) and electro-valves. Since it is well known that higher flow rates lead to greater resistance changes, it is worth noting that the low flow rate applied sacrifices response intensity but has the advantage of enabling lower power consumption and working under more realistic experimental conditions. The effect of the ambient moisture on the sensor’s performance was also assessed by using a controller evaporator mixer (Bronkhorst High-Tech B.V., The Netherlands) for humidifying the atmosphere.

The sensor resistances were monitored using a multimeter (HP 34972A, Agilent, USA), registering the resistance changes induced by different concentrations of gases. Specifically, the sensors were exposed to given concentrations of target gases for 5 min and subsequently stabilized for 15 min under dry airflow. Several concentrations of gas species were applied by performing successive dilutions with pure dry air, and the sensing responses were defined as (Δ*R*/*R*_0_) expressed in percentage. Where R_0_ is defined as the resistance level of the sensor in the air, while Δ*R* corresponds to the resistance changes obtained over 5 min of gas exposure.

### Optical Gas Sensing Measurements

Samples for carrying out UV–vis and PL experiments were prepared by dispersing 5 mg of the sample (Mo_6_ or Mo_6_@Graphene) in 1 mL of acetonitrile and sonicated for 10 min. Subsequently, the suspensions were deposited by drop-casting on an acid-treated quartz substrate (Mo_6_ sample) and an alumina substrate (Mo_6_@Graphene sample) placed on a hotplate at 50 °C. Then, the samples were placed inside a screw cap cuvette and fixed with a small piece of commercial Blu-Tack (Figure S17). The samples were purged for 20 min with Ar, air, and NO_2_ (balanced with pure synthetic air) before each in situ measurement. The measurements with NH_3_ were done by gas diffusion of 10 μL of ammonium hydroxide solution (28% NH_3_ wt, Sigma-Aldrich) placed at the bottom of the cuvette. The amount of gaseous NH_3_ was calculated from Henry’s law (H^cp^ = 0.58 M/Pa at 298.15 K) considering the equilibrium concentration of ammonia in solution (*k*_b_ = 1.8 × 10^–5^).

### Stability Studies of the Samples toward Selected Gases

The stability of the nanomaterials was monitored by registering the powder X-ray diffraction patterns and Raman spectra of both nanomaterials (deposited as a thin film on glass substrates) before and after exposure to NO_2_ and NH_3_ gases. These measurements were done with 30 min of exposure to each gas in a 5 mL vial with a septum and following a similar methodology as that described in the previous subsection.

### Computational Details

The calculations were conducted with the Gaussian 09 program suite. Density functional theory was applied with the PBE0 functional.^62,63^ Relativistic electron core potentials from the Stuttgart group and its associated basis sets^64^ were used to represent the molybdenum^65^ and iodine atoms^66^ and augmented in the case of Mo by an f polarization function (Mo/α = 1.043)^67^ and in the case of I, with a d polarization function (I/α = 0.289).^68^ The 6-31G(d,p) basis set was used to represent the remaining atoms (O, N, and H) of the molecular systems. The geometry optimizations were performed in the gas phase without any symmetry constraint, followed by analytical frequency calculations to confirm that a minimum has been reached. Zero-point energies were included in BE calculations, and the BEs were calculated using BE = *E*_cluster-molecule_ – [*E*_cluster_ + *E*_molecule_].

The *trans-* and *cis*-[Mo_6_I^i^_8_(OH)^a^_4_(H_2_O)^a^_2_] isomers were built as representative models for adsorption sites due to the fact that water and hydroxo positioning around the octahedral metal framework cannot be inferred from the X-ray diffraction structure. The guess geometries of the gas–cluster adducts were raised considering a random positioning of the gas analyte with respect to all the cluster ligands (water, hydroxo, and iodide) of the two cluster isomers.

## Supporting Information Available

Additional experimental and computational results, including photographs of the device and setups (PDF)
