# Supplementary material for: Octahedral Molybdenum Iodide Clusters Supported on Graphene for Resistive and Optical Gas Sensing
Source: ACS Appl Mater Interfaces. 2022 Dec 13;14(51):57122–32. doi: 10.1021/acsami.2c15716 (PMC9801382; doi:10.1021/acsami.2c15716)
Supplement: Supplementary file 1 — am2c15716_si_001.pdf [file am2c15716_si_001.pdf]

Supporting Information

Octahedral Molybdenum Iodide Clusters Supported on Graphene for Resistive and Optical Gas Sensing

Juan Casanova-Chafer, [a*] *Rocio Garcia-Aboal,* [b] *Pedro Atienzar,* [b] *Marta Feliz,* [b*] *and Eduard Llobet* [a]

[a] MINOS Research Group, Department of Electronics Engineering, Universitat Rovira i Virgili, 43007 Tarragona, Spain.

[b] Instituto de Tecnología Química, Universitat Politècnica de València - Consejo Superior de Investigaciones Científicas (UPV-CSIC), Avd. de los Naranjos s/n, 46022 Valencia, Spain.

*** Corresponding authors**:

Juan Casanova-Chafer, juan.casanova@urv.cat

Marta Feliz, mfeliz@itq.upv.es

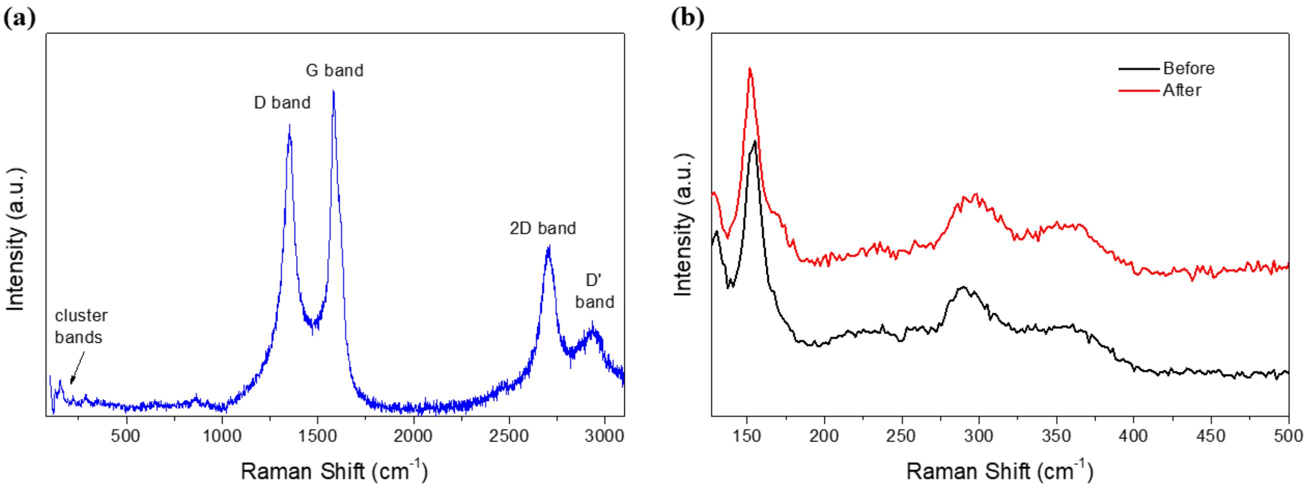

Figure S1. **(a)** Raman spectrum of Mo_6@Graphene hybrid. The bands identified at low Raman shifts are characteristic bands of the $\{Mo_6I^i_8\}^{4+}$ cluster core material. **(b)** Raman region of the Mo_6 sample measured before (black line) and after (red line) thermal treatment (60 °C). All the spectra were obtained at 514 nm.

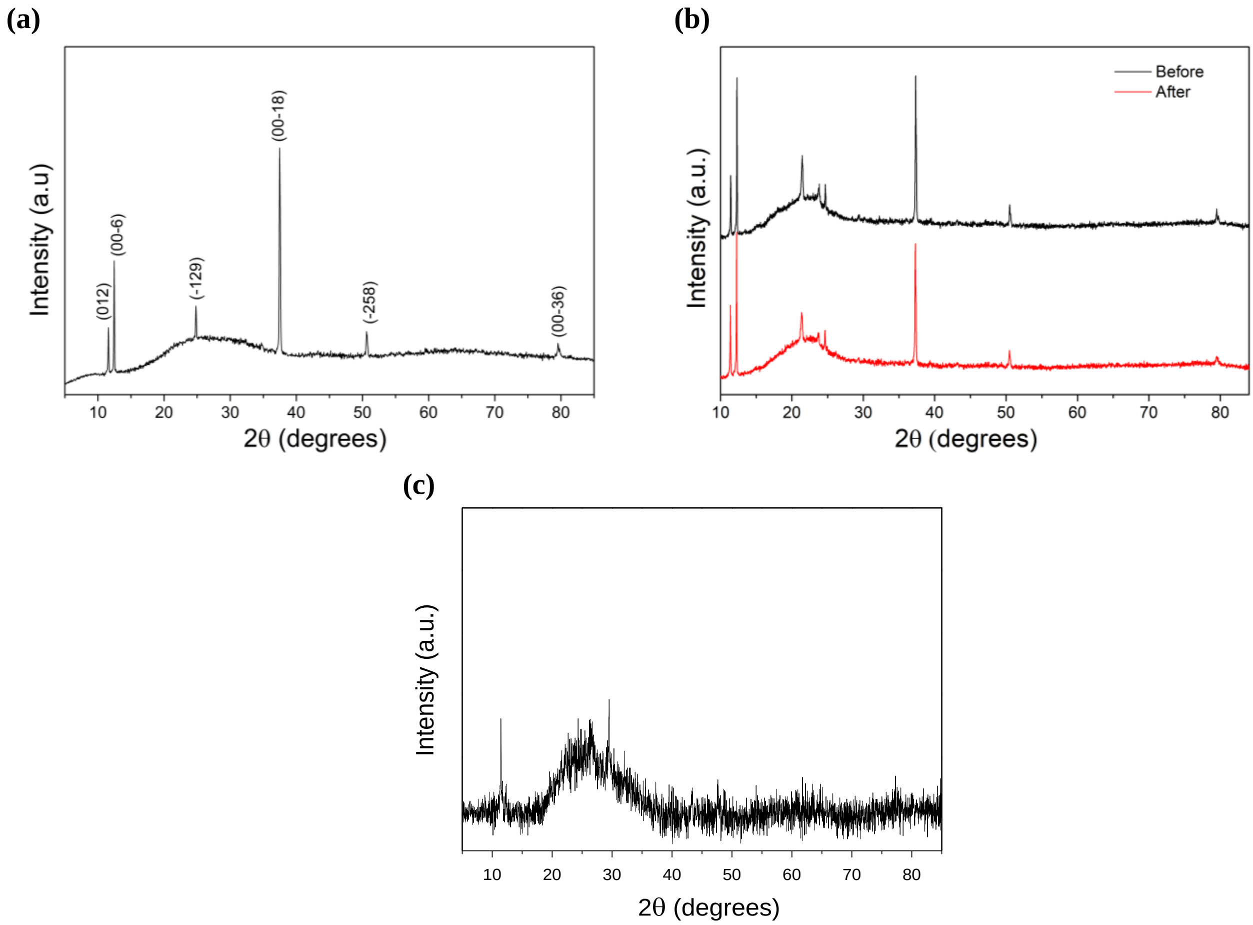

Figure S2. **(a)** Powder X-ray diffractogram and lattice planes of the Mo_6 material. (**b**) Powder X-ray patterns of the Mo_6 sample before (black line) and after (red line) thermal treatment (60 °C). It can be noticed that thermal treatment does not damage or degrade the Mo_6 cluster. The two extra peaks present between 22-25 degrees are associated to the lattice planes of the Mo_6 material, which are present due to the reorientation of the crystalline phase deposited onto the glass surface. **(c)** Powder X-ray pattern of the Mo_6@Graphene material.

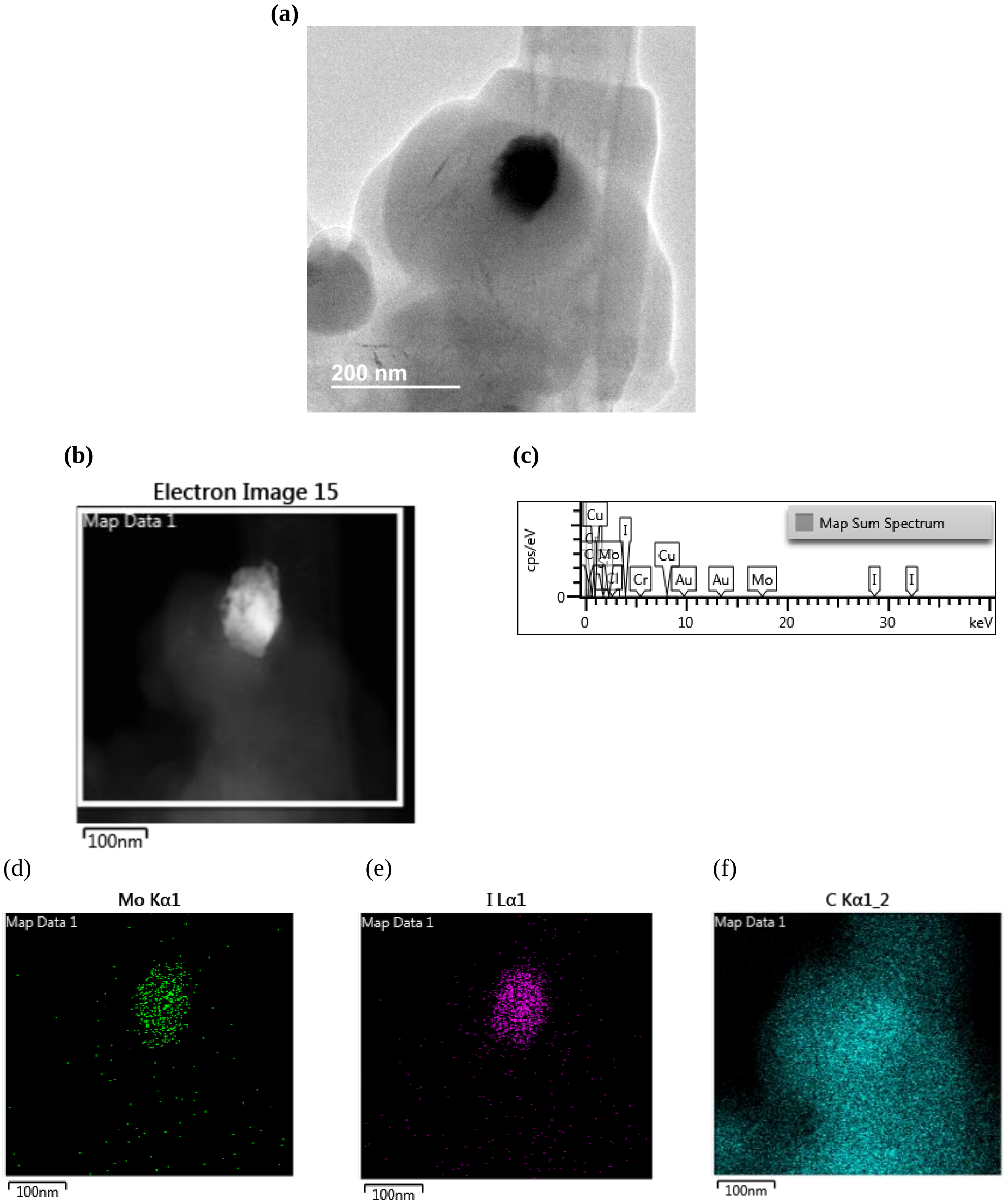

Figure S3. (**a**) HR-TEM image of the Mo_6@Graphene sample, (**b**) dark-field STEM image, **(c)** map sum spectrum, and EDS **(d)** Mo, **(e)** I, and **(f)** C mapping analyses of the Mo_6@Graphene sample.

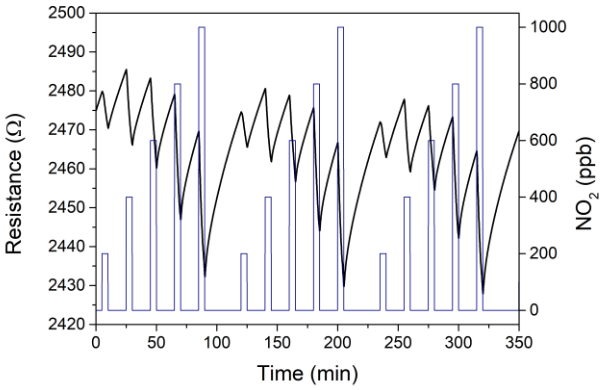

Figure S4. Dynamic response for a bare graphene sensor when detecting NO_2 dosed from 250 to 1000 ppb in dry conditions at room temperature. (Resistance: black line and left y-axis; Gas concentration: blue line and right y-axis).

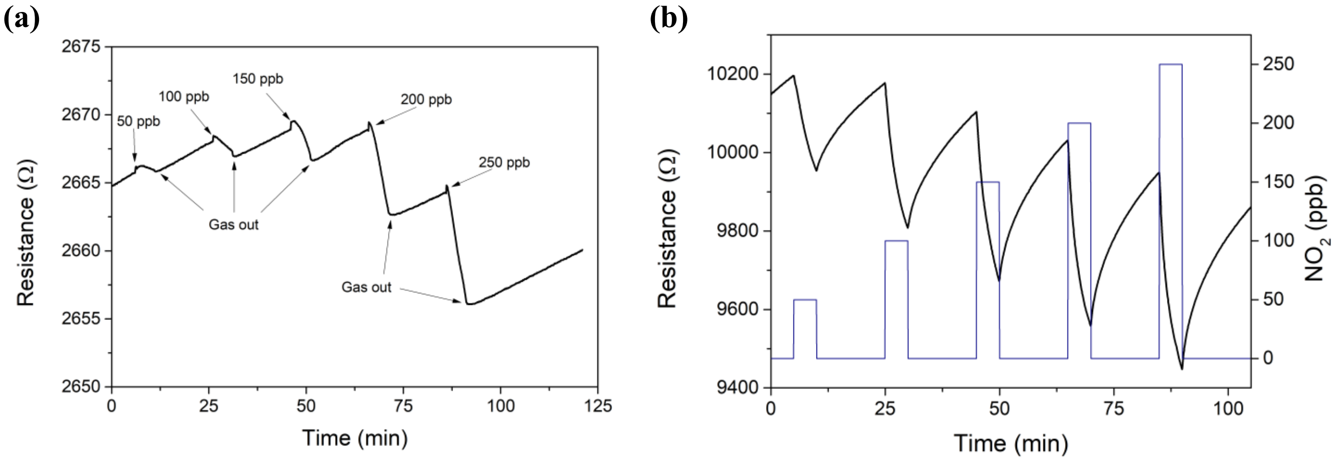

Figure S5. Electrical responses towards NO_2 (50-250 ppb) under dry conditions for a bare graphene sensor (**a**) and under humid conditions for a Mo_6@Graphene layer (**b**). All the measurements were conducted at room temperature. (Resistance: black line and left y-axis; Gas concentration: blue line and right y-axis).

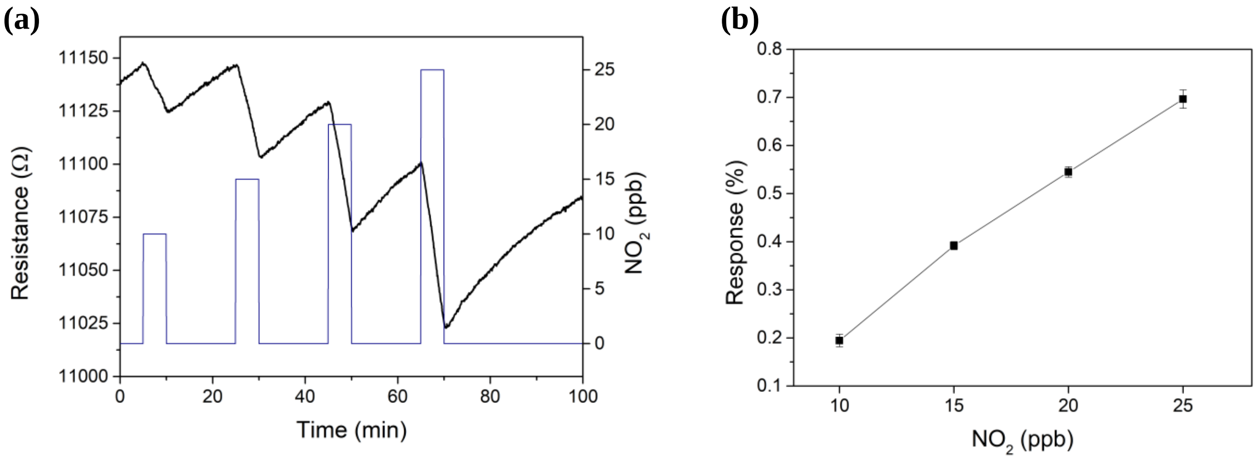

Figure S6. (**a**) Example of the resistance changes obtained for Mo6@Graphene layer when detecting NO_2 at trace levels. (Resistance: black line and left y-axis; Gas concentration: blue line and right y-axis). (**b**) Calibration curve for a Mo6@Graphene sample for NO_2 concentrations dosed from 10 to 25 ppb under dry conditions and at room temperature. It is worth highlighting that experimental LOD and LOQ values obtained are highly dependent on the sensing conditions applied. In this case, the trace levels detection was performed by keeping the same experimental conditions as the other measurements, but further optimization can be performed (*i.e.*, adjusting the exposure and recovery times or modifying the flow rate) for improving the LOD and LOQ values.

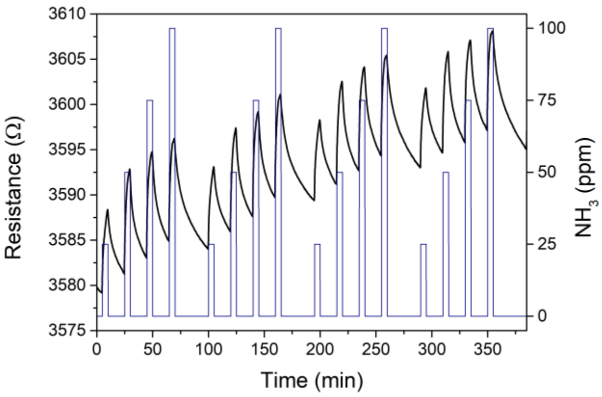

Figure S7. Example of the resistance changes when detecting NH_3 using a bare graphene nanomaterial. The measurement was conducted in dry conditions and at room temperature. (Resistance: black line and left y-axis; Gas concentration: blue line and right y-axis).

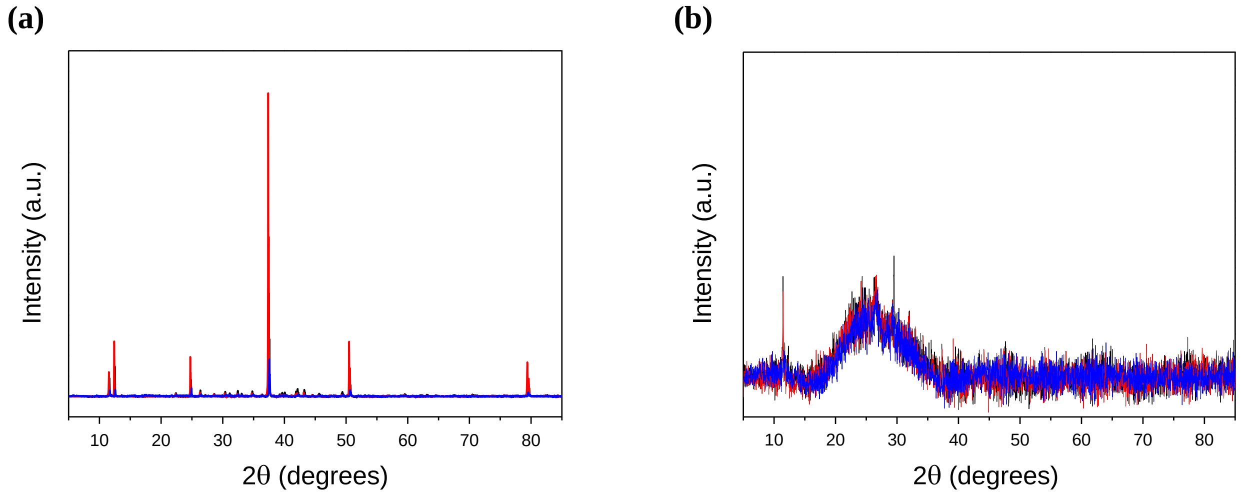

Figure S8. **(a)** Powder X-ray diffractograms of the Mo_6 sample before (black line) and after 30 minutes of NH_3 (red line) and NO_2 (blue line) exposure. **(b)** Powder X-ray patterns of the Mo_6@Graphene sample before (black line) and after 30 minutes of NH_3 (red line) and NO_2 (blue line) exposure. These figures show the high stability of the sensitive layer developed when detecting toxic gases.

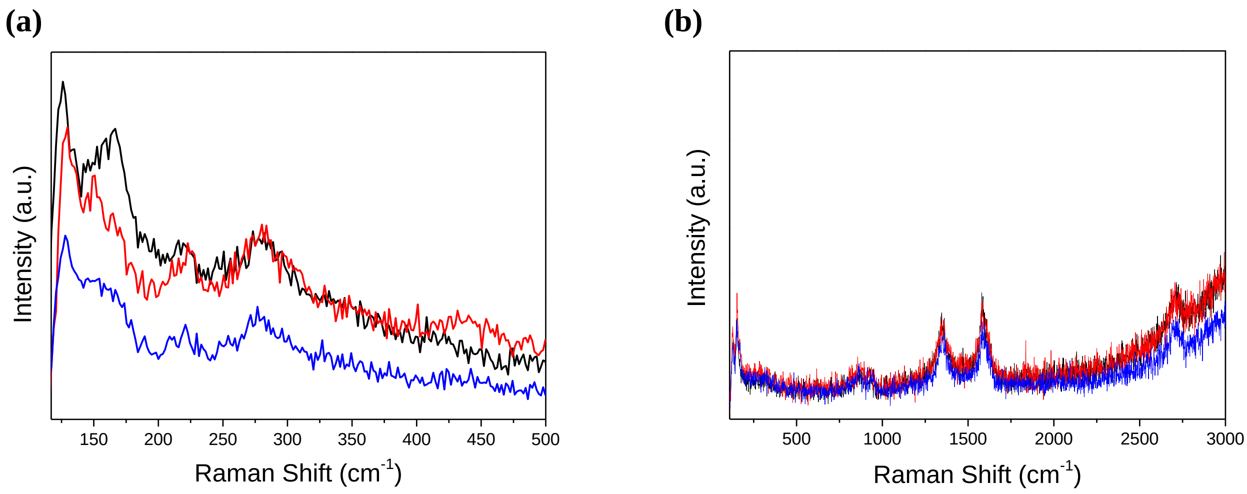

Figure S9. **(a)** Raman spectrum of Mo_6 sample before (black line) and after 30 minutes of NH_3 (red line) and NO_2 (blue line) exposure. **(b)** Raman spectrum of Mo_6@Graphene sample before (black line) and after 30 minutes of NH_3 (red line) and NO_2 (blue line) exposure. These figures show the high stability of the sensitive layer developed when detecting toxic gases.

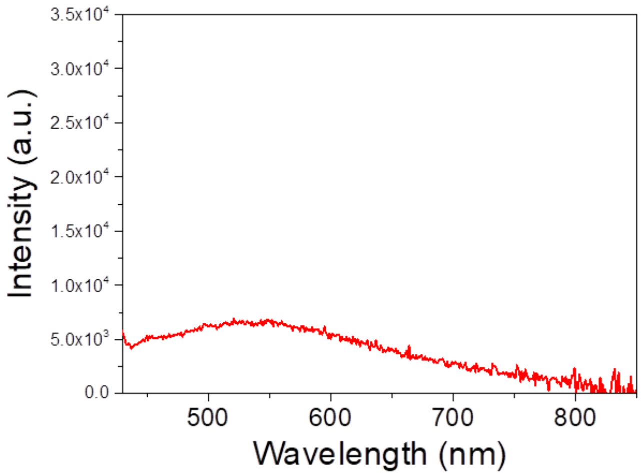

Figure S10. Room-temperature PL spectra of a thin film of graphene deposited in a quartz substrate (λ_{ex}=320 nm) and exposed under Ar.

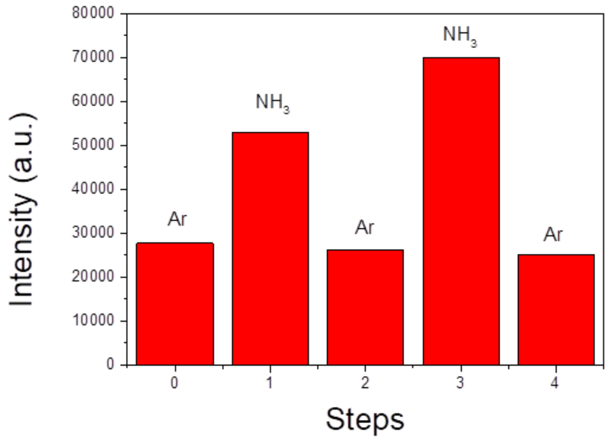

Figure S11. Bar diagram showing the emission intensity maxima changes (λ_{max} = 725 nm) of a Mo_6@Graphene layer deposited on a quartz substrate after exposure to some consecutive cycles to Ar and NH_3 gases, recorded at room-temperature (λ_{ex} = 320 nm).

(a)

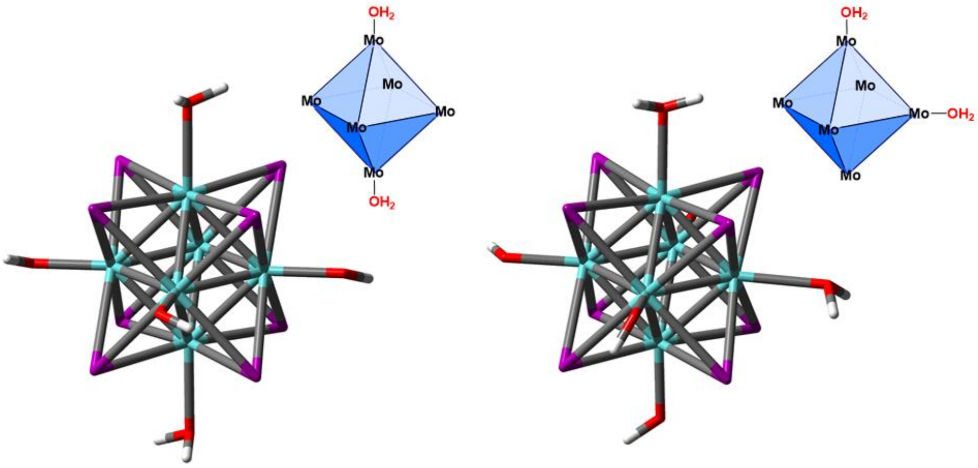

(b)

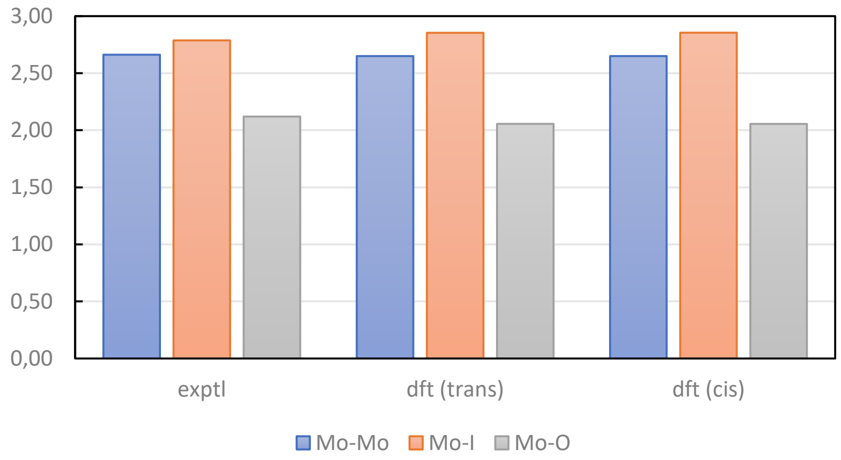

Figure S12. **(a)** DFT optimized geometries of the *trans*- and *cis*-$[Mo_6I^i_8(OH)^a_4(H_2O)^a_2]$ models with their respective simplified representations as insets (halogen and hydroxo ligands are omitted for clarity). **(b)** Comparison between DFT and experimental (single crystal X-ray diffraction) Mo–Mo, Mo-I^i and Mo–O^a interatomic distances (average values, in Å) for the *trans* and *cis*-$[Mo_6I^i_8(OH)^a_4(H_2O)^a_2]$ models.

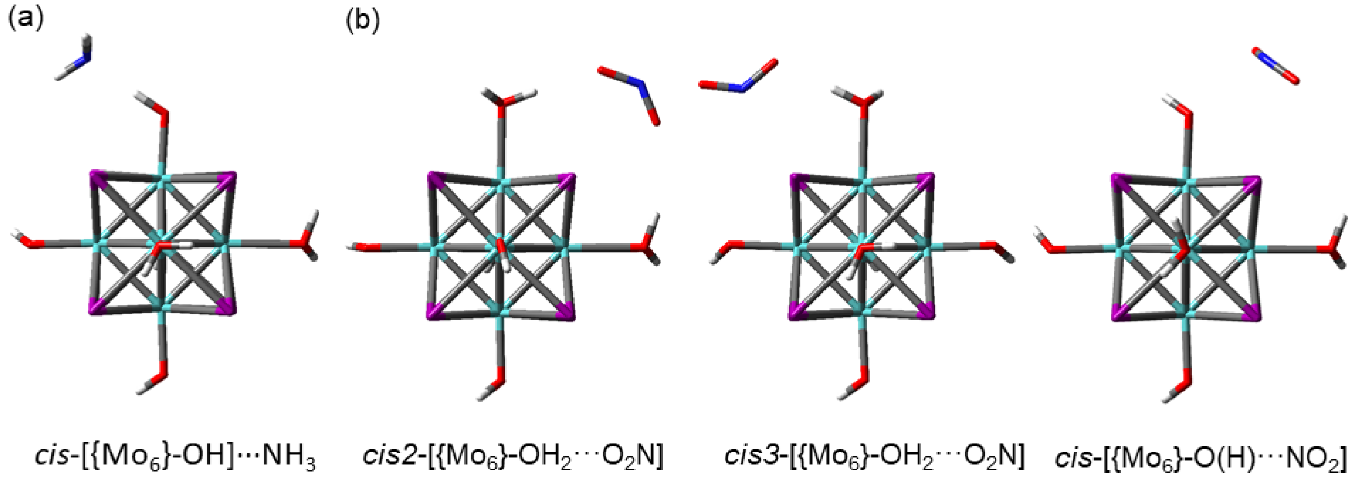

Figure S13. Representation of additional cluster···NH_3 **(a)** and cluster···NO_2 **(b)** structures, encompassing *trans*- and *cis*-$[Mo_6I^i_8(OH)^a_4(H_2O)^a_2]$ configurations.

Table S1. Binding energies (BEs) and relative energies (E_{rel}) of each adduct with respect the most stable cluster adduct with NH_3 and NO_2.

**Cluster-molecule adduct**	**BE (kcal/mol)**	**E_{rel} (kcal/mol)**
trans-[{Mo_6}-$OH_2\cdots NH_3$]	-17.8	0.0
cis-[{Mo_6}-$OH_2\cdots NH_3$]	-17.4	1.0
cis-[{Mo_6}-OH]$\cdots NH_3$]	-8.7	10.1
trans-[{Mo_6}-$OH_2\cdots O_2N$]	-6.8	0.0
cis-[{Mo_6}-$OH_2\cdots O_2N$]	-6.5	0.3
cis2-[{Mo_6}-$OH_2\cdots O_2N$]	-5.8	1.6
cis3-[{Mo_6}-$OH_2\cdots O_2N$]	-5.6	1.2
cis-[{Mo_6}-O(H)$\cdots NO_2$]	-5.8	1.7

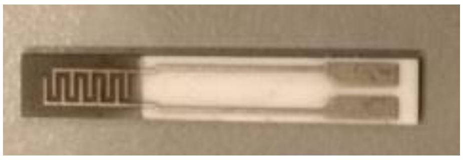

Figure S14. A layer of molybdenum clusters decorating graphene deposited onto an alumina substrate with platinum screen-printed electrodes.

(a) **(b)**

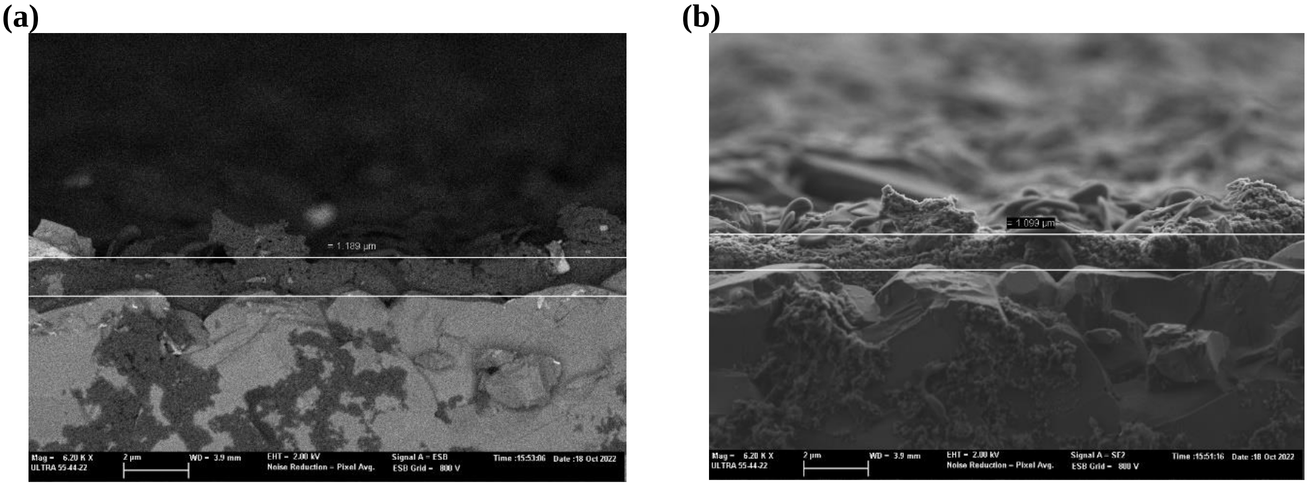

Figure S15. FESEM images acquired with two different detectors: Energy selective Backscattered (ESB) detector, **(a)**, and Secondary Electron (ES2) detector **(b)** of the cross-section of the Mo_6@graphene layer deposited onto the alumina substrate electrode. The parallel lines indicate thickness of the Mo_6@graphene layer.

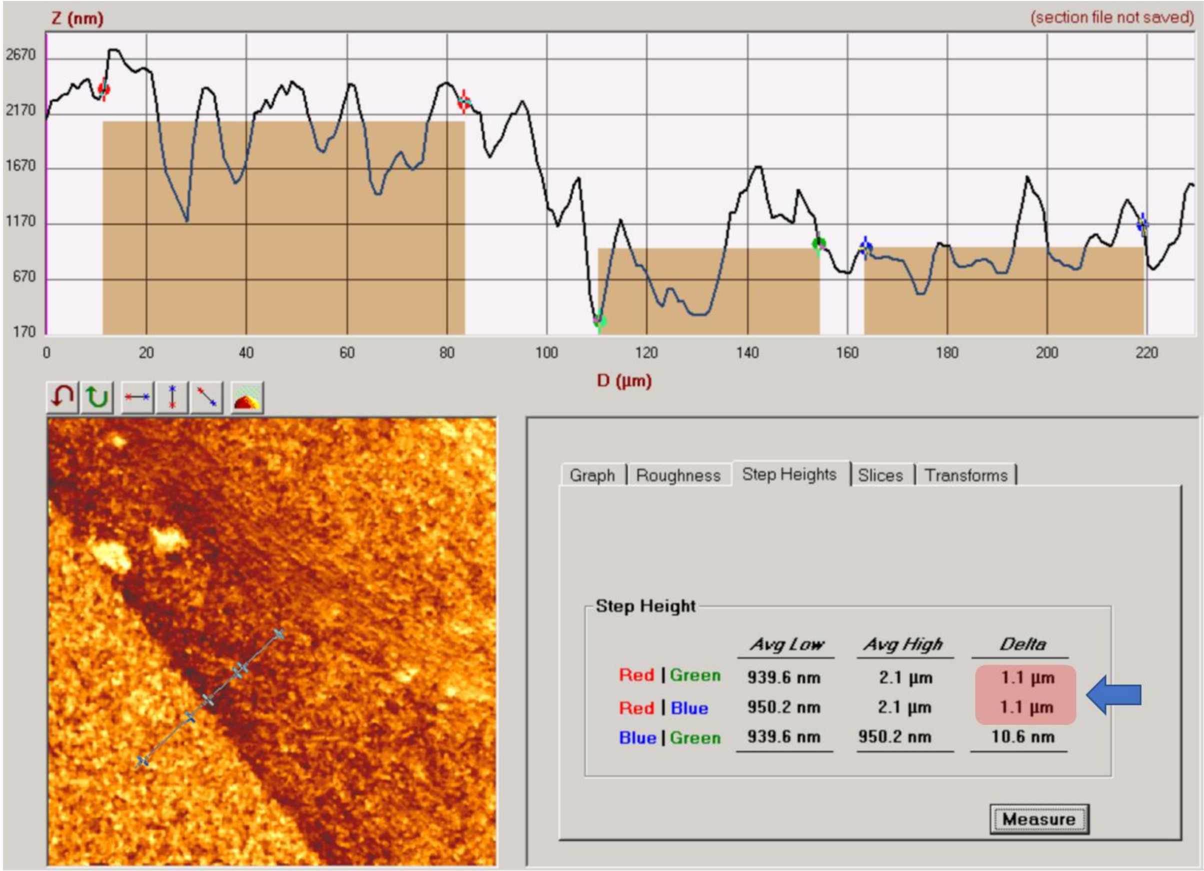

Figure S16. Layer thickness measured with an optical profilometer of a Mo_6@graphene layer deposited onto the alumina substrate electrode. The arrow indicates the value of the thickness.

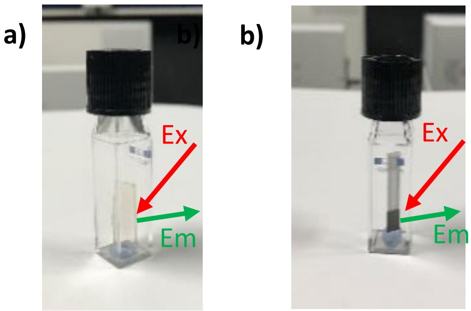

Figure S17. Thin film of Mo_6 deposited on a quartz substrate **(a)** and Mo_6@Graphene onto an electronic board **(b)**. The arrows indicate the excitation and emission for monitoring.
